# Supplementary material for: Quantifying impacts of the COVID-19 pandemic through life-expectancy losses: a population-level study of 29 countries
Source: Int J Epidemiol. 2021 Sep 26;51(1):63–74. doi: 10.1093/ije/dyab207 (PMC8500096; doi:10.1093/ije/dyab207)
Supplement: dyab207_Supplementary_Data [file dyab207_supplementary_data.zip › dyab207-suppl_data/ije-2021-04-0547-File007.pdf]

Quantifying impacts of the COVID-19 pandemic through life expectancy losses: a population-level study of 29 countries

Supplementary File 1

José Manuel Aburto[‡,§,*], Jonas Schöley[*,†], Ilya Kashnitsky[*], Luyin Zhang[‡,¶]
Charles Rahal[‡,§], Trifon I. Missov[*], Melinda C. Mills[‡,§],
Jennifer B. Dowd[‡,§], Ridhi Kashyap[‡,§,†]

[‡] *Leverhulme Centre for Demographic Science and Department of Sociology, University of Oxford*
[§] *Nuffield College, University of Oxford*
[*] *Interdisciplinary Centre on Population Dynamics, University of Southern Denmark*
[†] *Max Planck Institute for Demographic Research, Rostock*
[¶] *St. Cross College, University of Oxford*

6th August, 2021

Country/Region	All cause death counts # Groups 2019	2020	Miss. 2020	Open-age 2019	2020	Covid deaths Latest report 2020	Notes
Austria	19	19	0	90	90	31/12/2020	COVID death counts not available by sex
Australia	5	5	0	85	85	31/12/2020	Coarse ages.
**Belgium**	19	19	0	90	90	31/12/2020	
Bulgaria	19	19	0	90	90	/	COVID death counts unavailable.
Canada	4	4	0	85	85	22/09/2022	Coarse ages.
**Switzerland**	19	19	0	90	90	31/12/2020	
**Chile**	20	20	0	95	95	31/12/2020	No data for 2015.
**Czech Republic**	19	19	0	90	90	31/12/2020	
**Germany**	15	15	0	95	95	31/12/2020	No data for 2015.
**Denmark**	21	21	0	100	100	31/12/2020	
Estonia	19	19	0	90	90	/	COVID death counts unavailable.
**Spain**	19	19	0	90	90	31/12/2020	
Finland	19	19	0	90	90	/	COVID death counts not available by sex.
**France**	21	21	0	95	90	31/12/2020	
**England & Wales**	106	20	0	100	90	25/12/2020	Pre-2020 data from ONS.
Northern Ireland	19	19	0	90	85	/	Last 2 weeks with 7 age-groups. COVID deaths not available by sex.
**Scotland**	20	21	0	90	95	31/12/2020	
Greece	19	19	0	90	90	31/12/2020	No data 2015.
Croatia	19	19	0	90	90	/	
**Hungary**	19	19	0	90	90	31/12/2020	
Israel	8	8	0	80	80	28/12/2020	Coarse ages.
Iceland	19	19	0	90	90	/	
**Italy**	22	19	0	100	90	29/12/2020	Missing weeks.
South Korea	5	5	0	85	85	28/06/2020	Coarse age grouping. COVID deaths after 2020-06-28 not available by sex.
Lithuania	19	19	0	90	90	/	COVID death counts unavailable.
Luxembourg	19	19	0	90	90	/	Low number of younger deaths and small pop sizes produced atypical shape of hazard with PCLM. COVID death counts unavailable.
Latvia	19	19	0	90	90	/	Low number of younger deaths and small pop sizes produced atypical shape of hazard with PCLM. COVID death counts unavailable.
**Netherlands**	19	19	0	90	90	27/12/2020	
**Norway**	21	21	0	100	100	30/12/2020	
New Zealand	3	3	0	80	80	/	Coarse age grouping. COVID deaths not available by sex.
**Poland**	19	19	0	90	90	/	COVID deaths not available.
**Portugal**	19	19	0	90	90	31/12/2020	
Russia	19	/	53	90	/	/	No data for 2020. COVID deaths not available.
Sweden	19	19	0	90	90	/	COVID deaths not available by sex.
**Slovenia**	19	19	0	90	90	31/12/2020	
Slovakia	19	19	0	90	90	/	COVID deaths not available.
Taiwan	21	21	1	100	100	10/08/2020	Missing weeks for 2020. COVID deaths incomplete.
USA	11	11	0	85	85	26/12/2020	Pre-2020 data from CDC, aggregated to 2020 age grouping.

Table S1: Data quality of raw all-cause and Covid-19 death data. Note: Source data retrieved 2021-05-25 from HMD-STMF, CoverAge-DB, UK-ONS, and US-CDC. All cause death counts from STMF unless stated otherwise in the notes. Countries noted in italics were excluded from analysis. Countries in bold are part of COVID-19 analysis. 'Miss.' refers to latest reported data in 2020.

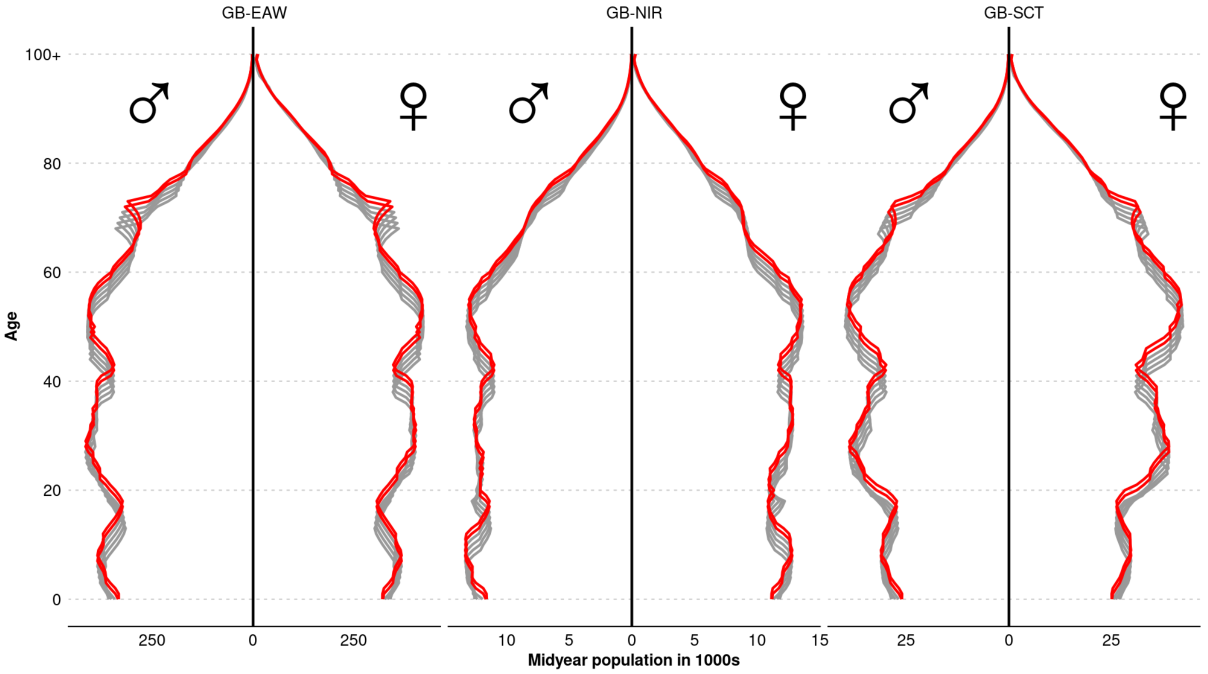

Figure S1: Midyear population by age 2015-2018 as reported by the Human Mortality Database and own projections for 2019-2020 for England Wales, Scotland and Northern Ireland.

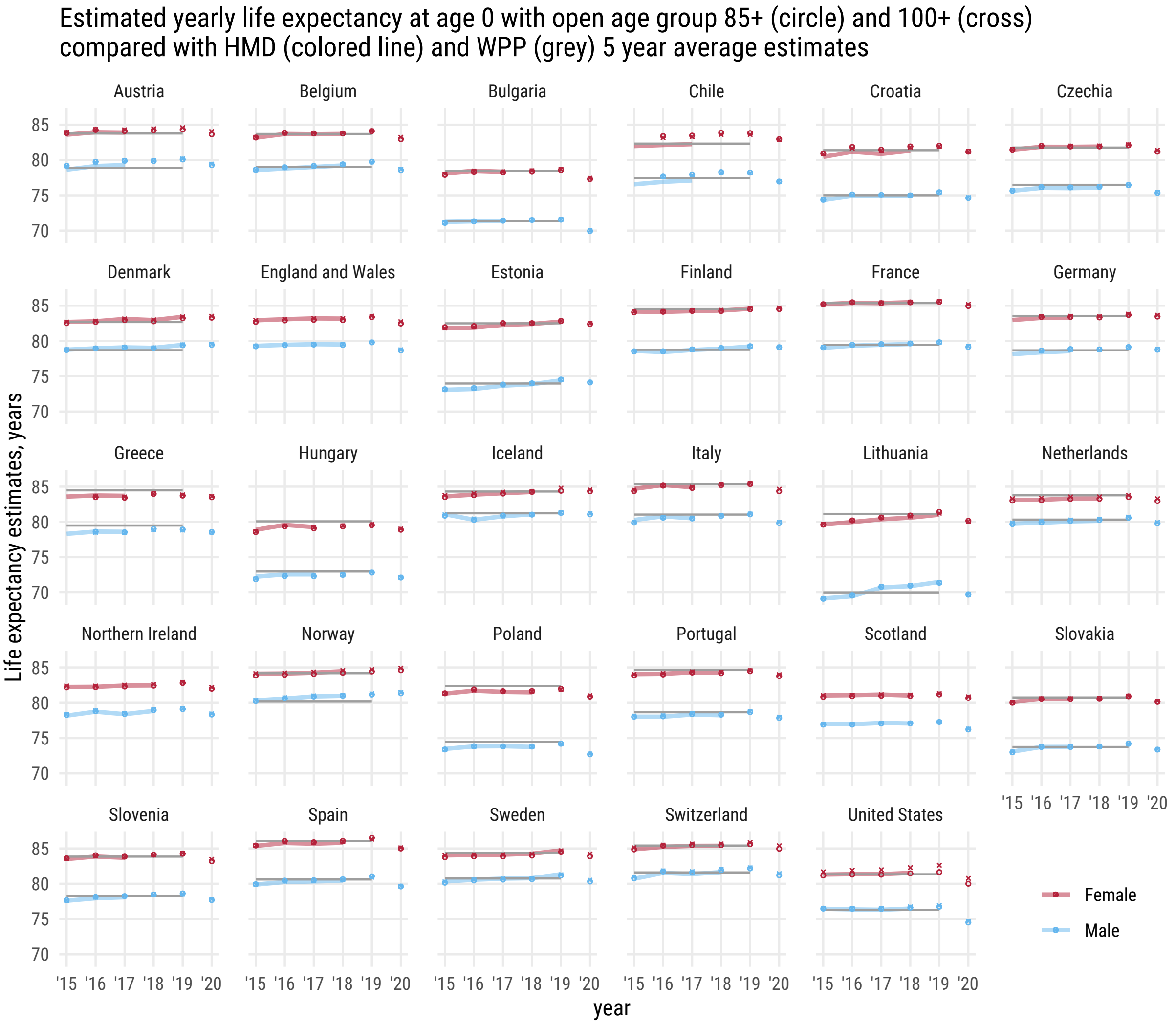

Figure S2: Comparison of estimates of life expectancy by closing life tables at 85+ and 100+ with those published by the United Nations and the Human Mortality Database in the period 2015-19, or the most updated year available.

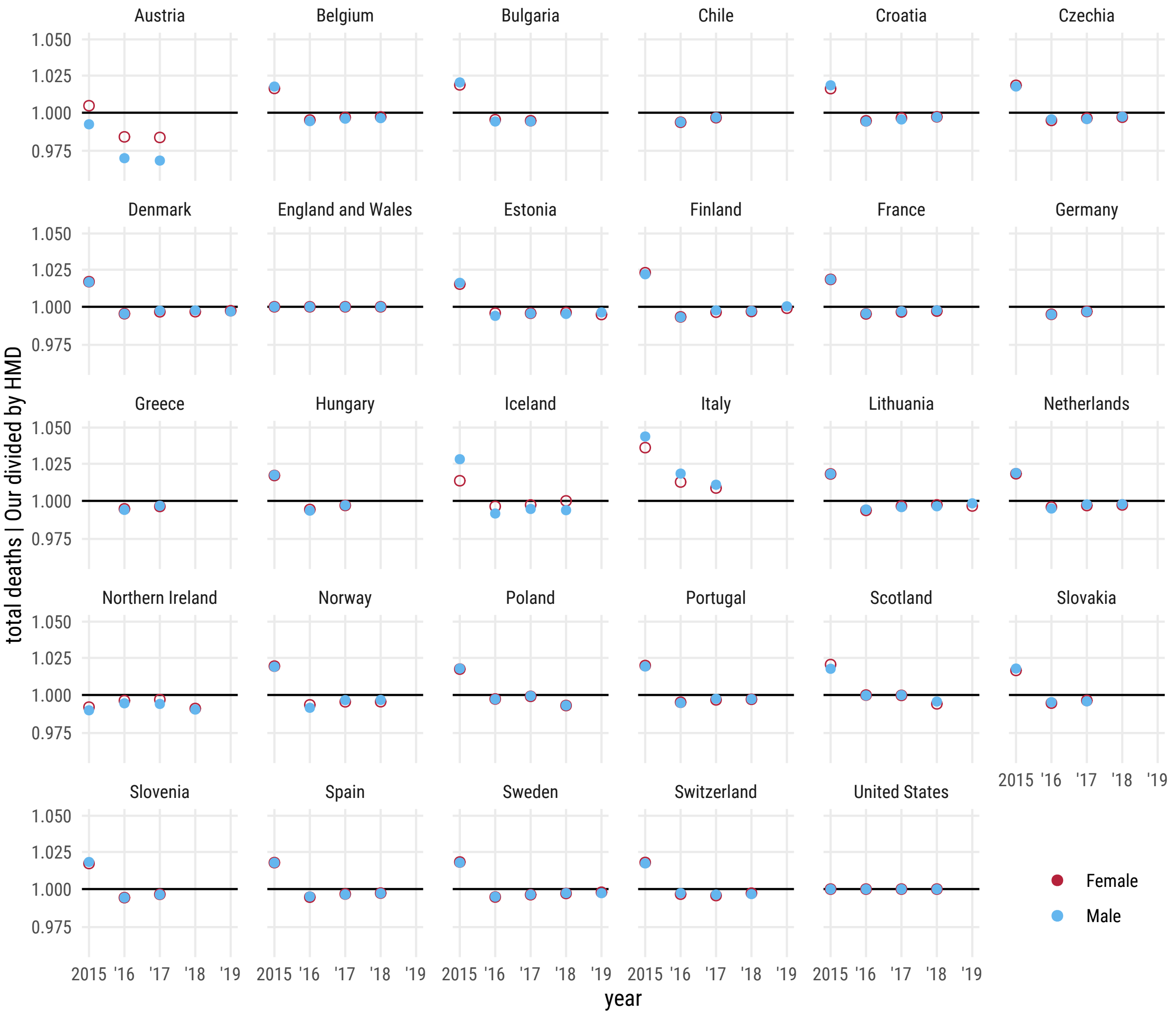

Figure S3: Comparison of death counts between the Short Term Mortality Fluctuations dataset and the Human Mortality Database. Note: for discrepancy in 2015 see Methods section on ISO-week formats.

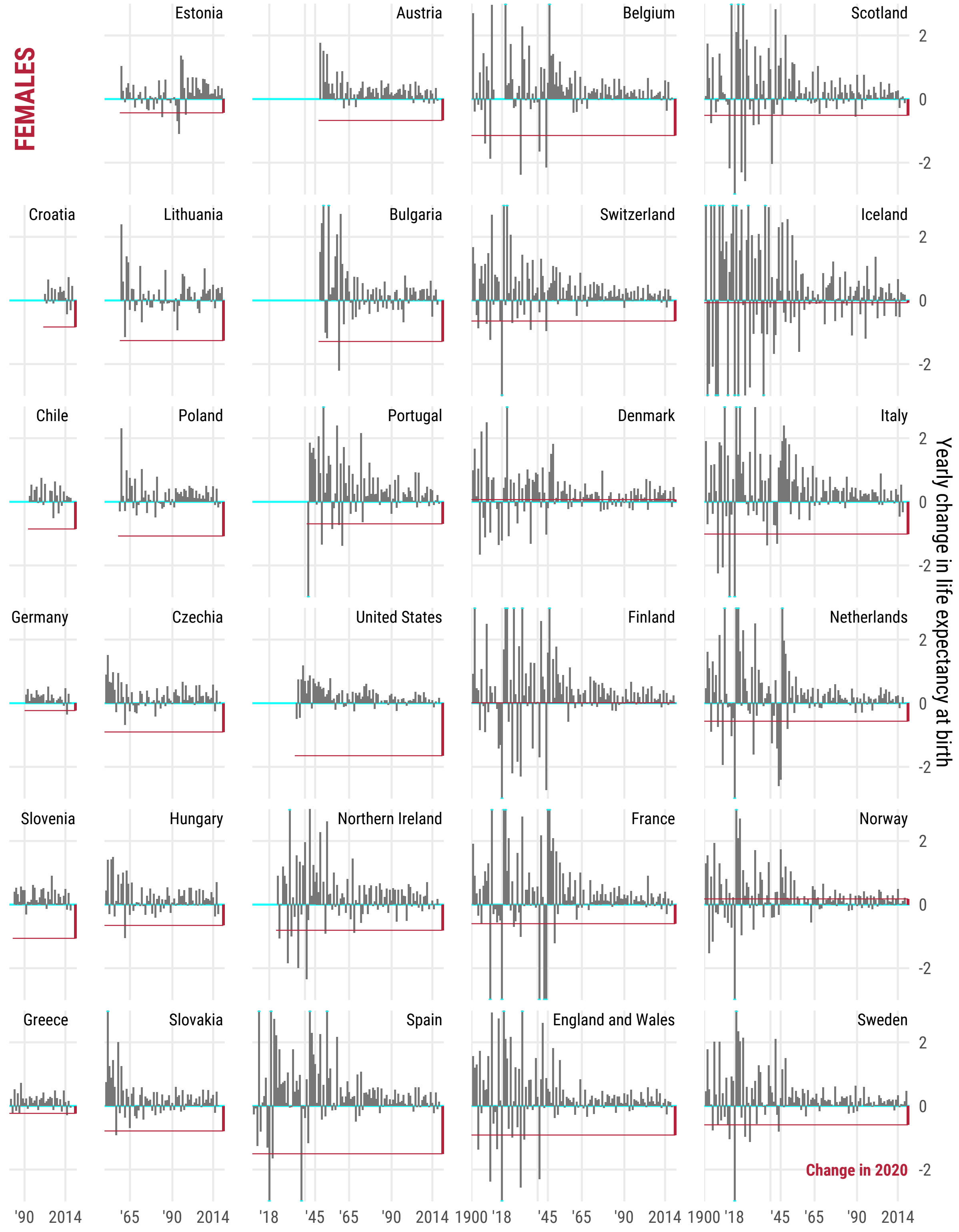

Figure S4: Annual change in life expectancy at birth since 1900 or earliest year available in the Human Mortality Database for females. The straight red indicates the change from 2019 to 2020, with the bars representing year-on-year change.

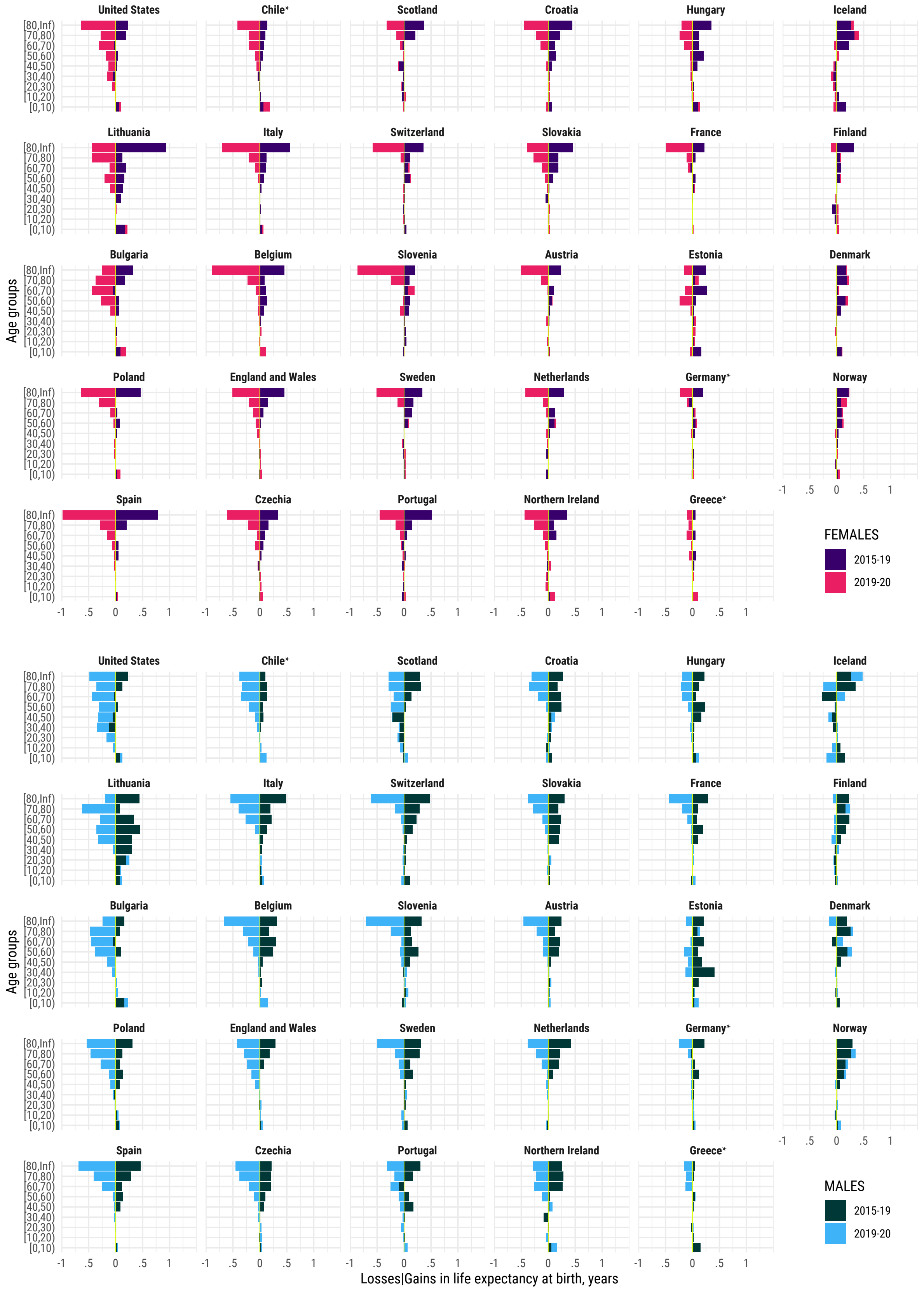

Figure S5: Contributions (in years) to changes in life expectancy at birth from 2015 to 2019, and from 2019 to 2020, attributable to mortality in different 10-year age groups by country and sex. Note: Positive values indicate gains in life expectancy, negative values indicate reductions in life expectancy. *Estimates for Chile and Germany were available from 2016.
